# *Plasmodium falciparum *variant STEVOR antigens are expressed in merozoites and possibly associated with erythrocyte invasion

**DOI:** 10.1186/1475-2875-7-137

**Published:** 2008-07-23

**Authors:** Ayman Khattab, Insa Bonow, Nadine Schreiber, Michaela Petter, Christel Schmetz, Mo-Quen Klinkert

**Affiliations:** 1 Department of Molecular Medicine, Bernhard-Nocht Institute for Tropical Medicine, Hamburg, Germany; 2 EM Laboratory, Bernhard-Nocht Institute for Tropical Medicine, Hamburg, Germany; 3 Malaria Research Laboratory, Department of Bacteriology and Immunology, Haartman Institute, University of Helsinki, Haartmaninkatu 3, FIN-00014 Helsinki, Finland

## Abstract

**Background:**

*Plasmodium falciparum *STEVOR proteins, encoded by the multicopy *stevor *gene family have no known biological functions. Their expression and unique locations in different parasite life cycle stages evoke multiple functionalities. Their abundance and hypervariability support a role in antigenic variation.

**Methods:**

Immunoblotting of total parasite proteins with an anti-STEVOR antibody was used to identify variant antigens of this gene family and to follow changes in STEVOR expression in parasite populations panned on CSA or CD36 receptors. Immunofluorescence assays and immunoelectron microscopy were performed to study the subcellular localization of STEVOR proteins in different parasite stages. The capacity of the antibody to inhibit merozoite invasion of erythrocytes was assessed to determine whether STEVOR variants were involved in the invasion process.

**Results:**

Antigenic variation of STEVORs at the protein level was observed in blood stage parasites. STEVOR variants were found to be present on the merozoite surface and in rhoptries. An insight into a participation in erythrocyte invasion was gained through an immunofluorescence analysis of a sequence of thin slides representing progressive steps in erythrocyte invasion. An interesting feature of the staining pattern was what appeared to be the release of STEVORs around the invading merozoites. Because the anti-STEVOR antibody did not inhibit invasion, the role of STEVORs in this process remains unknown.

**Conclusion:**

The localization of STEVOR proteins to the merozoite surface and the rhoptries together with its prevalence as a released component in the invading merozoite suggest a role of these antigens in adhesion and/or immune evasion in the erythrocyte invasion process. These observations would also justify STEVORs for undergoing antigenic variation. Even though a role in erythrocyte invasion remains speculative, an association of members of the STEVOR protein family with invasion-related events has been shown.

## Background

Of the four species causing human malaria, *Plasmodium falciparum *is the most fatal aetiologic agent. Its ability to invade erythrocytes of all ages leads to high blood levels of parasitaemia. It exports proteins to the erythrocyte surface, facilitating cytoadherence to endothelial receptors and microvascular sequestration, thereby causing abnormal function of the affected tissues [1].

The multicopy *var *gene family encoding variant surface antigens known as *P. falciparum *erythrocyte membrane protein 1 (PfEMP1) is the best characterized exported protein that undergoes antigenic variation (reviewed in [2]). Other variant antigens with speculated roles in antigenic variation also belong to multigene families, namely the *rif *(repetitive interspersed family), *stevor *(subtelomeric variable open reading frame) and *Pfmc-2TM *multicopy gene families [3]. In carrying a signal peptide and a Pexel/VTS motif (*Plasmodium *export element/vacuolar transport signal) [4,5], these proteins are potentially exported to the host cytoplasm and membrane. The presence of a hypervariable loop domain flanked by two predicted transmembrane domains is also suggestive of surface exposure. Expression of antigenically distinct RIFIN variants in different parasite strains and life cycle stages was recently observed [6]. Transcription studies demonstrated that the expression of *stevor *and *Pfmc-2TM *gene families is clonally variant and undergoes switching [7], but confirmation of antigenic variation at the protein level is lacking.

STEVORs are reported in sporozoites and gametocytes [8], and in Maurer's clefts (MC) of trophozoites and schizonts [9]. MC are flattened vesicular structures beneath the infected erythrocyte (IE) membrane and may translocate parasite proteins to the erythrocyte surface [10]. Because of their expression and unique location in different parasite stages, these proteins probably have multiple functions.

The *Plasmodium *parasite undergoes three invasive stages during its life cycle. Invasion of erythrocytes by merozoites in iterative cycles causes the clinical manifestations of the disease, including fever, anaemia and coma [11]. Merozoite proteins are thus considered vaccination targets, and strategies include blocking maturation of infective merozoites, exit from host erythrocytes, and invasion of new erythrocytes. Synthetic 20-mer peptides from one STEVOR protein and antibodies thereof were shown to inhibit merozoite invasion of erythrocytes [12].

Here, STEVOR proteins were identified in merozoites and protein release during erythrocyte invasion was demonstrated. Implication of multicopy variant antigens in invasion adds to the complexity of the process and opens up new avenues for research on blocking the malarial life cycle.

## Methods

### Parasite strains and culture

The *Plasmodium falciparum *strains used in this study were NF54 and Gb337 [13]. The strains were cultured in O^+ ^human erythrocytes at 5% haematocrit in RPMI-1640 medium supplemented with 0.5% Albumax II (Gibco, Carlsbad, CA, USA), 2% human AB serum (PAA, Pasching, Austria), 200 μM hypoxanthine (Sigma, St. Louis, MO, USA) and 20 μg/mL gentamycin (Gibco).

### Enrichment of CSA and CD36 phenotypes

The NF54^CSA ^and NF54^CD36 ^parasite phenotypes were generated by the selection of chondroitin sulfate A (CSA) and CD36 binding NF54 parasites on immobilized CSA and C32 melanoma cells, respectively. The resulting parasite phenotypes were used via immunoblotting to examine the effect of parasite selection and phenotypic changes on levels of STEVOR expression and STEVOR variant types.

### Lysed infected erythrocytes preparation and immunoblotting

Erythrocytes infected with *P. falciparum *ring stages were synchronized twice, 4 h apart, by sorbitol treatment (Sigma, St. Louis, MO, USA) [14] and grown to high parasitaemia (≈ 10%) of mostly late trophozoite stages. Late-stage IE were enriched to a parasitaemia of about 90 – 95% by magnet-activated cell sorting (MACS). Enriched IE were resuspended in 500 μl PBS, counted, lysed by the addition of saponin to a final concentration of 0.15% and kept on ice for 15 min. Lysed IE were collected by centrifugation for 5 min at 13,000 rpm at 4°C, the pellet was washed twice in PBS followed by centrifugation and the proteins from the final pellet were extracted by boiling in 1 × SDS sample buffer for 10 min. A fraction of the protein extract equivalent to 3 × 10^7 ^IE/lane was loaded onto SDS-polyacrylamide gels and separated proteins were transferred to Trans-Blot transfer medium membrane (Bio-Rad, Hercules, CA, USA). Equal loading was also confirmed by Ponceau S staining. The membrane was probed with mouse anti-STEVOR antibodies, as previously described [15], namely anti-PFA0750w, -PFL2610w, -MAL13P1.7 or -PFC0025c (each at 1:3000). These antibodies were previously incubated with unrelated His-fusion protein blotted to membranes to absorb out the anti-His antibodies. Reactivity was visualized by goat anti-mouse IgG coupled to horseradish peroxidase (1:30 000; Pierce, Rockford, IL, USA). Blots were developed by the enhanced chemiluminescent (ECL Western blot analysis system)-based detection according to manufacturer's instructions (GE Healthcare, Life Sciences, Bucks, UK).

### Immunofluorescence assays and co-localization

For indirect IFA, parasites were cultured and synchronized as described above and allowed to grow for one cycle before samples were processed. IE representing the ring, trophozoite and schizont stages were smeared on glass slides, air-dried and fixed for 5 min with ice cold 100% methanol. Gametocyte stage parasites were prepared as previously described [16] with the slight modification that no N-acetyl-D-glucosamine was added and fixed as described above. Slides from different stages were incubated at room temperature for 2 h with mouse anti-STEVOR antibodies (1:500) or a mix of mouse anti-STEVOR antibodies (1:500) and rabbit anti-MSP1 antibody (1:200). Binding of the primary antibodies was visualized by Alexa 488-conjugated goat anti-mouse (1:1000; Molecular Probes) when anti-STEVOR antibodies were solely used, whereas a mixture of Alexa 594-conjugated goat anti-mouse (1:400; Molecular Probes) and Cy2-conjugated goat anti-rabbit antibodies (1:400; Dianova, Hamburg, Germany) were used when combining the analysis of both anti-STEVOR and anti-MSP1 primary antibodies. Cell nuclei were visualized by DAPI (4',6-diamidino-2-phenylindole) (5 μg/ml; Roth, Germany). Slides were mounted on MOWIOL (Calbiochem, San Diego, CA, USA), viewed and captured using a Leica fluorescence microscope with the help of OpenLab software. Pre-immune sera were used to replace the corresponding anti-STEVOR antibodies for the negative control slides.

### Immunoelectron microscopy

Late stage parasites were enriched by MACS and fixed using a mixture of 1% paraformaldehyde and 0.025% glutaraldehyde in cacodylate buffer for 1 h at 4°C, embedded in LR white resin with the accelerator (London Resin Company, London, UK) and sectioned with a Reichert Ultracut E microtome (Leica, Germany). Ultrathin sections were incubated for 15 min in PBS, blocked for 1 h at room temperature using PBS/BSA (10%) and incubated with anti-STEVOR antibody (anti-PFL2610w) (1:10 000) for 1 h at 37°C and overnight at 4°C. After four washes with PBS/BSA, the sections were incubated with rat anti-mouse antibody (1:5000, Dianova, Hamburg, Germany) for 45 min at 20°C. Sections were washed four times with PBS/BSA and were treated with gold (10 nM) conjugated Protein A (Department of Cell Biology, School of Medicine, University of Utrecht, NL) at a dilution of 1:70 for 45 min at 20°C. Thereafter, sections were washed five times using PBS and stained with 2% uranyl acetate for 5 min and Reynolds lead citrate for 1 min for contrast. After drying, the grids were then viewed on Philips CM 10 TEM. For negative controls, the primary antibody was replaced by the pre-immune serum.

### Capture of invasive merozoites

*Plasmodium falciparum *3D7 parasite was cultured and synchronized as described above and allowed to grow for one cycle before the onset of the experiment. Parasites in the second cycle were grown to the late schizont stage and parasite thin slides were prepared every 30 min over a 4 h window (estimated time of invasion ± 2 h). Slides representing the invasion process, as assessed by Giemsa staining for merozoite attachment to erythrocyte surfaces, were analysed in IFA using anti-PFL2610w antibody as a representative anti-STEVOR antibody.

### Antibody invasion inhibition assay

Serum and Protein G-purified total IgG generated against the PFL2610w STEVOR variant and from a naïve mouse were used in the inhibition assays. Anti-MSP1 antiserum served as a positive control. The invasion inhibition assays were performed in duplicates one cycle after synchronization by inoculation of fresh erythrocytes with IE (at 0.5% parasitaemia and 2% haematocrit) in a 100 μl volume in 96-well flat-bottomed tissue culture plates [17]. Wells with only uninfected cells served as blanks, those with both infected and uninfected but no antibodies as controls. Sera constituted 1, 3 or 10% of the total assay volume. Either serum or purified IgG samples (at final concentrations of 0.1, 0.3 or 1 mg/ml) were added at assay set up, prior to reinvasion. Parasites and antibodies were incubated for 24 h, thereafter [^3^H]hypoxanthine (GE Healthcare, Life Sciences, Bucks, UK) was added (1 μCi/well). After 16 h, cells were harvested onto glass filters and incorporated radioactivity determined as counts per minute (cpm). Calculations were made using the formula:

$$\%\text{ inhibition}=\text{1}00-\frac{\left[\left(\text{cpmin test well}-\text{cpm in blank well}\right)\times \text{1}00\right]}{\text{cpm in control well}-\text{cpm in blank well}}$$

## Results

### Antigenic variation and parasite phenotype

Multiple *stevor *transcripts in a given parasite population have previously been identified [18]. Synthesis in a single cell of 2 to 3 copies of full-length transcripts has also been reported [19]. To date it is unknown whether multiple *stevor *transcripts are correlated with the expression of multiple STEVOR proteins. Evidence for clonal variation and gene switching in *stevor *genes transcription was recently described [20]; however, occurrence of antigenic variation at the protein level is still lacking. By analogy with specific *var *gene expression being related to distinct binding phenotypes, we questioned if such a phenomenon existed for STEVORs. To follow changes in STEVOR expression, two different parasite lines NF54^CD36 ^and NF54^CSA^, each with binding phenotypes specific for either CD36 or CSA, respectively were used. The CD36 selected line was expected to represent a set of multiple clones (population), at least in vitro in the absence of immune pressure. However, we believe that each one was capable of CD36 binding, based on the knowledge that there is no a strict link of this phenotype to a single *var *gene, unlike the CSA-binding phenotype for which the *var2CSA *gene is responsible. Parasite lines NF54^CD36-R ^and NF54^CSA-R^, following loss of their original binding phenotypes through subsequent rounds of culture under no selection pressure were included. Anti-STEVOR antibodies generated against four different STEVOR variants, whose genes were cloned from the *P. falciparum *3D7 parasite were available for study [21]. Each of these four antibodies recognized the corresponding recombinant protein and the other three proteins indicating that the individual antibody cross reacts with other STEVOR proteins (data not shown). Presumably the cross reactivity is due to the common conserved domains carried on the recombinant proteins. Cross reactivity with uninfected erythrocyte proteins or members of a closely related protein family such as that of the RIFIN family was ruled out by immunoblot analyses using total lysates prepared from uninfected erythrocytes or an array of representative recombinant RIFIN proteins [22,23].

**Figure 1:**
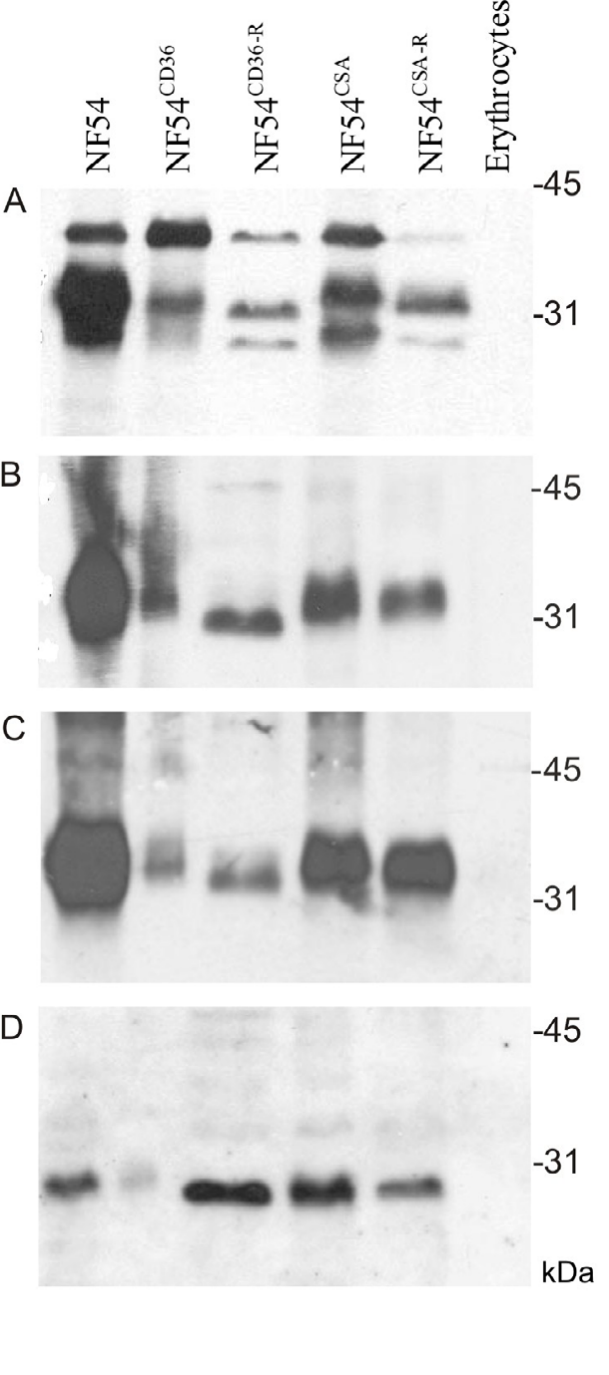
**STEVOR expression and parasite binding phenotype**. Immunoblots of parasite-IE extracts and human uninfected erythrocytes were probed for STEVOR variations using (A) anti-PFL2610w, (B) anti-PFC0025c, (C) anti-MAL13P1.7 or (D) anti-PFA0750w antibodies. Relevant molecular mass markers are shown.

Upon analysis of the differentially selected parasite lines in immunoblots, considerable differences in band intensities (taken as a measure of the expression level of STEVOR variants) were observed compared to the parent parasite NF54. Using the anti-PFL2610w antibody to probe the lysed IE, three different STEVOR variants with molecular masses of 28, 32 and 40 kDa in the unselected parent line NF54 were observed, the 32 kDa variant being the most abundant (Figure 1A, lane 1). After selection for CD36 binding, the 28 kDa variant in NF54^CD36 ^almost disappeared while the 32 kDa variant showed reduced band intensities, in contrast the 40 kDa band which increased in intensity (lane 2). After loss of CD36 binding, a different level of STEVOR expression was observed, whereby all 3 STEVOR variants reappeared in intensities different from both NF54 and NF54^CD36 ^parasites (lane 3).

Similar changes in STEVOR expression profiles were observed for the NF54^CSA ^and the NF54^CSA-R ^parasite lines. Relative to the parent parasite NF54, the 28, 32 and 40 kDa variants were present in NF54^CSA ^parasites, except that the 32 kDa band was expressed at a lower level in the parent line (lane 4). Loss of the CSA-binding phenotype led to a dramatic decrease to an almost undetectable level for the 28 and 40 kDa variants, whereas the expression level of what appears to be the 32 kDa variant remained (lane 5). No STEVORs were detected in uninfected erythrocytes analysed in parallel (lane 6). Recognition of protein bands of different sizes and intensities was confirmed with anti-PFC0025c, anti-MAL13P1.7 and anti-PFA0750w antibodies (Figure 1B, C and 1D, respectively).

### Detection of STEVOR proteins in merozoites

**Figure 2:**
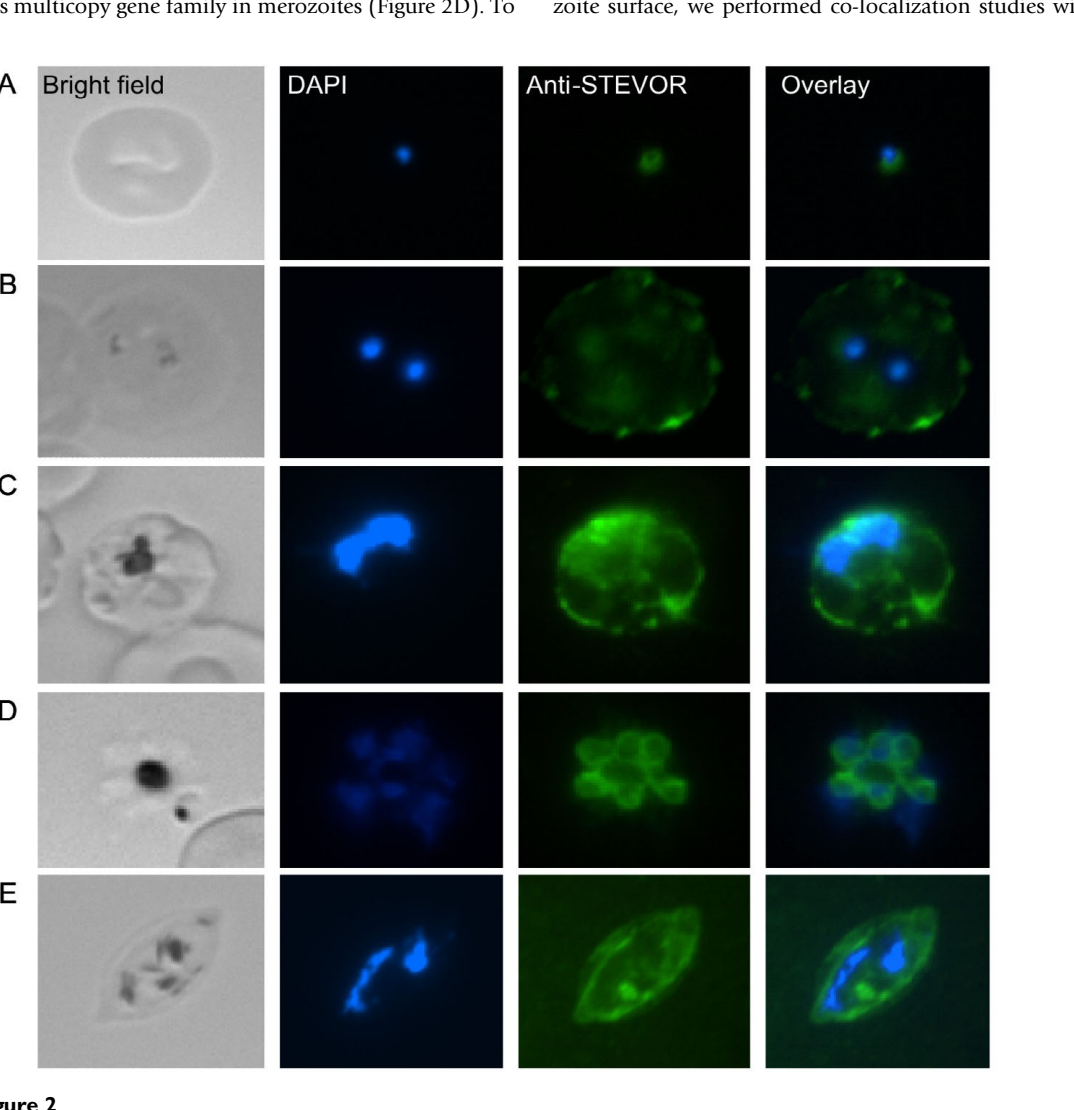
**STEVOR proteins in *P. falciparum *blood stage parasites**. IFA images of parasite strain Gb337, cultured from a field isolate in (A) early ring, (B) ring, (C) trophozoite, (D) schizont and (E) gametocyte stages are visualized using anti-PFL2610w antibody. Bright field images (first column), parasite nuclei stained with DAPI (second column), STEVORs visualized with Alexa 488 (third column) and overlay of nuclei and STEVOR protein staining (fourth column) are shown.

Anti-STEVOR peptide antibodies were used to demonstrate its presence in MC in trophozoite- and schizont-IE [24]. Other workers have localized STEVORs in the plasma membrane of gametocyte-IE and in discrete foci within the sporozoites [25]. In our IFA experiments, we show perinuclear localization of STEVOR in the early post-invasion ring stage using polyclonal anti-PFL2610w antibody (Figure 2A). Late ring stage staining was evidenced by light punctuate fluorescence signals, in addition to a discrete rim staining associated with the IE surface (Figure 2B). Trophozoite-IE gave a similar pattern, but stronger overall staining was observed (Figure 2C). Dual staining with anti-PfSBP1 antibodies confirmed STEVORs to be localized in MC (data not shown). The most striking observation, never seen before, is the detection of this multicopy gene family in merozoites (Figure 2D). To complete the picture of stage specificity, fluorescence staining performed in gametocytes revealed STEVOR proteins internally, in the gametocyte membrane and also in the IE membrane (Figure 2E).

### Co-localization of STEVORs with the merozoite surface protein (MSP) 1

**Figure 3:**
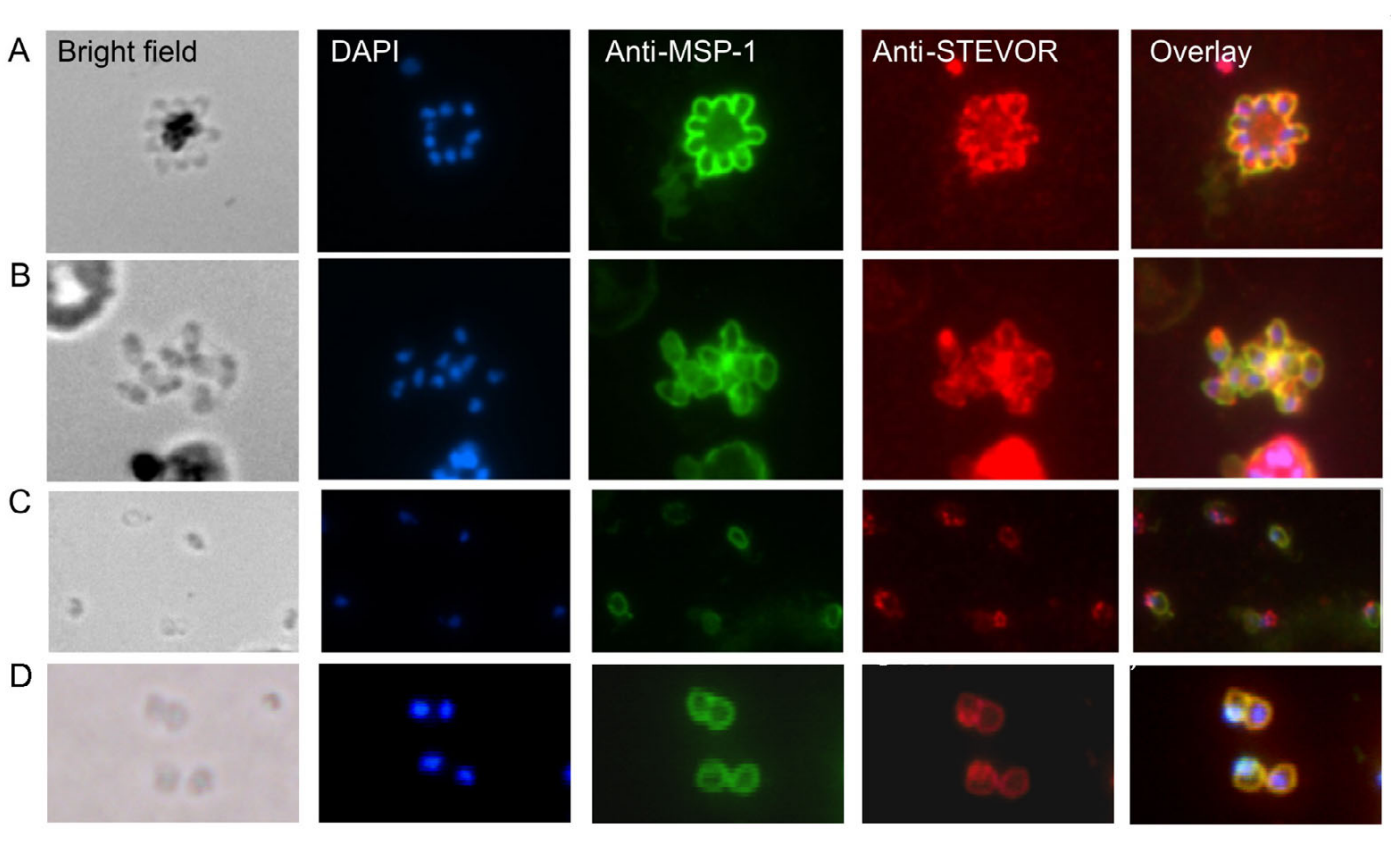
**Co-localization of STEVOR proteins with MSP-1**. Fluorescence staining using anti-PFL2610w and anti-MSP-1 antibodies was analysed in (A) a *P. falciparum *schizont, (B) a ruptured schizont and (C and D) free merozoites. Bright field images (left column), DAPI stained parasite nuclei (second column), Alexa 488 stained MSP-1 (third column), Alexa 594 stained STEVOR proteins (fourth column) and the overlay of nuclei, MSP-1 and STEVOR proteins (fifth column) are shown.

To verify the close association of STEVORs with the merozoite surface, we performed co-localization studies with antibodies to MSP-1. The IFA experiments revealed a strict co-localization of STEVORs with MSP-1 in developing schizonts (Figure 3A) as well as in a ruptured schizont (Figure 3B), representatively shown for anti-PFL2610w antibodies. In addition to the surface fluorescence, anti-STEVOR antibody was seen to consistently and strongly stain a spot within the merozoite at the apical end, opposite of the nucleus. Further experiments to exclude the possibility that surface staining is due to membrane fragments arising from a rupturing schizont, naturally released free merozoites were prepared and stained with anti-STEVOR antibodies (Figure 3C and 3D). Fluorescence staining of both the surface of the merozoite as well as the additional spotted apical staining cap was evident. Overall, staining was strongest at the apical end, possibly reflecting greater abundance of STEVOR in this region of the merozoite.

### Location of STEVORs by immunoelectron microscopy (IEM)

**Figure 4:**
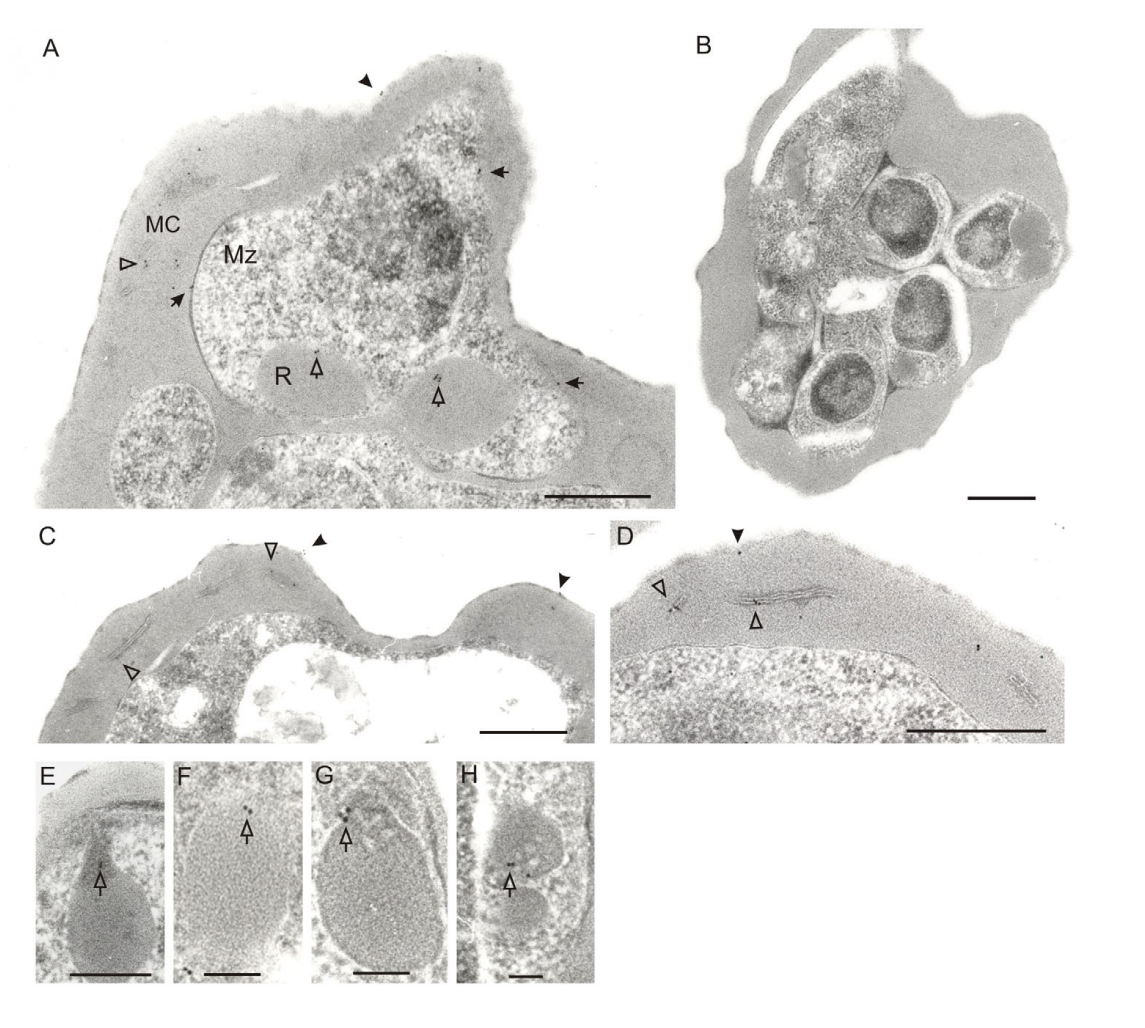
**Immunoelectron microscopic localization of STEVORs**. Ultrathin sections of schizont stage-IE were analysed using anti-PFL2610w antibody. Gold labelling is observed on the IE surface (A and C, black arrowheads), merozoite surface (A, black arrows), in MC (A, C and D, white arrowheads), rhoptries (A, white arrows) and rhoptry neck (E-H, white arrows). No gold particles are observed with pre-immune serum, shown for an IE containing developing merozoites (B). Scale bars are 0.5 μm for A and 1 μm for B-H.

To extend the IFA data, IEM was performed using anti-PFL2610w antibody. For the first time, gold particles were observed on the erythrocyte surface, associated both with electron dense knobs and the membrane bilayer (Figure 4A, C and 4D). Equally significant was the clear labelling in the electron-dense rhoptry organelles of the parasite (Figure 4A), sometimes specifically localized to the neck of the rhoptries (Figure 4E–H). Coherent staining on the surface of developing merozoites was evident (Figure 4A) as was the consistent labelling of MC (Figure 4A, C and 4D). No reactivity was seen with pre-immune serum (Figure 4B).

In general, a low density of gold particles was a characteristic on the IEM sections and this could be attributed to the high dilution of the antibody (1:10,000) used in the experiments or to the paucity of STEVOR proteins on the IEM sections.

### Implication of STEVORs in merozoite invasion

Here, IFA evidence for STEVOR protein expression in the merozoite was provided and their presence both on the surface and apically in the rhoptry were specifically shown in IEM. Prompted by these findings and based on knowledge that invasion is completed within ~30 seconds after merozoite release from schizonts, a role of STEVORs in the invasion process was explored.

**Figure 5:**
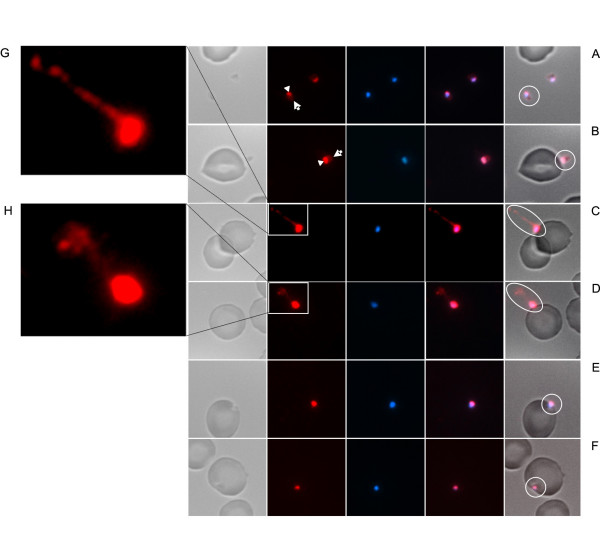
**STEVOR reactivity in merozoite invasion**. STEVORs are localized on merozoites (A) in free form, (B) docking, (C and D) invading, (E) invaginating and (F) entering an erythrocyte. Enlarged images (G and H) show staining of comet-like structures. White arrows and arrowheads indicate STEVORs on the merozoite surface and apex, respectively. White circles and ellipses highlight the invasive merozoite and the comet, respectively. Bright field images (first column), STEVOR proteins visualized with green Alexa 594 (second column), merozoite nuclei stained with blue DAPI (third column), overlay of STEVOR protein staining and nuclei (fourth column) and the overlay of bright field, STEVOR protein staining and nuclei (fifth column) are shown.

Slides representing invasion at progressive steps showed the presence of proteins from a variant multigene family in invading merozoites. In addition to surface staining of free merozoites with an apical focus (Figure 5A), we observe STEVOR labelling of a merozoite "docking" onto the erythrocyte surface (Figure 5B). Notably, STEVOR proteins were not only confined to the merozoite, but were also evident in association with a comet-like structure emanating from the invading merozoite (highlighted by ellipses) (Figure 5C and 5D). Whether STEVORs are being cleaved or are simply released together with other proteins involved invasion such as MSP-1 and AMA-1 remains to be studied. In subsequent steps involving invagination and entry, STEVORs continue to be present (Figure 5E and 5F, respectively).

### No inhibition of invasion by anti-STEVOR antibodies

**Figure 6:**
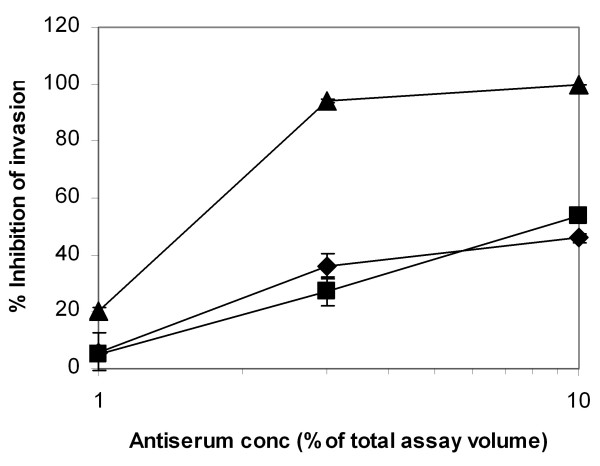
**Inhibition of erythrocyte invasion by *P. falciparum *merozoites**. Varying concentrations of antibody as a percentage of the total assay volume were used, as shown for anti-PFL2610w (■), pre-immune serum (◆) and anti-MSP-1 antibody (▲).

Prompted by the above observations, whether anti-STEVOR antibodies can block the erythrocyte invasion process was investigated. Anti-MSP-1 antiserum yielded 100% invasion inhibition when used at its maximal concentration (10%) in the assay. The same anti-PFL2610w antiserum concentration inhibited invasion by 54%, while pre-immune serum yielded a 46% inhibition (Figure 6). This finding suggests a non-specific interference of the sera with invasion. Despite the use of purified IgG fractions (up to 1 mg/ml), no significant adverse effects on merozoite invasion were observed. At this concentration, 36% invasion inhibition was observed with the test IgG sample, compared to 25% with pre-immune IgG.

## Discussion

In the asexual life cycle of the *P. falciparum *parasite, merozoites use different categories of proteins that function both intracellularly and extracellularly to invade new erythrocytes within 30 sec after their release from schizonts. Once inside, they develop within the parasitophorous vacuole (PV) and begin to export proteins to the erythrocyte cytoplasm and membrane, thereby modifying it. Exported proteins with structural functions are encoded by single copy genes, whose sequences hardly vary, such as KAHRP, MESA, PfEMP3 (reviewed in [26]) and PfSBP1 [27]. Those encoded by multigene families, such as PfEMP1, RIFIN and STEVOR carry hypervariable domains, which may have crucial roles in parasite survival.

Hypervariability in the coding sequence of STEVORs suggests that they undergo antigenic variation, even though data at the protein level are still lacking. In a study designed to assess gene transcription patterns in selected versus unselected 3D7 parasite populations by real time PCR [28], no change in the transcription pattern of *stevor *genes due to selection was observed, i.e. an erythrocyte population able to bind trHBMEC cell lines transcribes the same *stevors *at similar levels to those cells unable to bind. Another study using the limiting dilution method to create different NF54 parasite clones or populations showed that *stevor *transcription was clonally variant [29]. Here, evidence of antigenic variation in STEVORs at the protein level was presented, as seen by differences in levels of detected STEVOR proteins as well as presence or absence of one or more variants upon selection for a phenotype or loss of a phenotype. Such differences between unselected, selected and lost phenotype lines most probably reflect differences in parasite populations responsible for the expression of distinct STEVOR variants. In other words, selection of a binding phenotype seemed to be accompanied by an enrichment of major parasite population (s), their relative abundance to each other vary according to the major phenotype the parasite line represent. Accordingly, similar band patterns seen in Figure 1 were expected in both selected and unselected parasite line but dramatic changes in the expression level of STEVOR were observed upon selection. A loss of binding is also most likely to be accompanied by similar changes. Here, the antibodies not only recognized different STEVOR subtypes in different parasites but also identified STEVOR variants with different expression levels, subsequent to changes in their binding phenotypes. This expression data are discrepant with work by the former group but complement that of the latter group. The discrepancy could be attributed to the use of different parasite strains or to post-transcriptional control mechanisms leading to the selection of a distinct transcript for protein expression, and not another.

Earlier attempts to localize STEVORs in developing merozoites of *P. falciparum *schizonts using an anti-STEVOR peptide antibody failed [30], presumably because of low detection limits. The demonstration of STEVORs on the merozoite membrane was confirmed using an anti-MSP-1 antibody. Furthermore, IEM studies located the protein to the erythrocyte membrane, as expressed also by an epitope-tagged STEVOR in a transgenic NF54 parasite line [31]. STEVORs were additionally found in membranous structures in the erythrocyte cytosol. Many of these were in close vicinity to the surface membrane, which brings to mind the picture that these molecules are en route to the erythrocyte surface via the MC. Finally, fine mapping of STEVORs to the rhoptries is novel and never seen before for variant multigene family members. Examples of smaller protein families undergoing limited polymorphisms in rhoptries are reticulocyte binding-like proteins and RhopH1 encoded by the multicopy cytoadherence-linked asexual gene family (reviewed in [32]), with suggested or confirmed roles in host cell selection, binding, antigenic variation, PV formation and rhoptry biogenesis.

The report of STEVORs in what looked like a discharged merozoite coat is intriguing, opening up the possibility that STEVORs may play an important role in erythrocyte invasion. STEVORs could assist adhesion to the erythrocytes and/or protect the free merozoites from immune attack and could have also a supportive role in the release or cleavage of membranous structures from the merozoite. It is unlikely that the comet tail-like structures are an artefact caused by "smearing" of merozoite surface proteins during thin film preparation, since such structures are not seen around single "non-invading" merozoites, and comets pointing in different directions have also been observed on a same slide.

STEVORs remain associated with the merozoite during invagination even when comet tails (at least those in which STEVORs are present) are no longer detectable. This is suggestive of a further role after invasion, and unprocessed or rudimentary STEVORs of merozoite origin would explain the observed perinuclear IFA staining at early ring stages.

The failure to show invasion inhibition by a single polyclonal anti-STEVOR antibody does not diminish the biological significance of STEVORs in the parasite nor its vaccine candidacy. Conceivably, only antibodies generated against specific variants expressed in merozoites are inhibitory. Also, invasion inhibition is more likely achievable with monoclonal antibodies targetting crucial epitopes on the variable domain. Moreover, growth inhibition by purified human IgGs against putative vaccine antigens are known to be effective in a cell-mediated but not an antibody-dependent manner, exemplified by the merozoite surface glutamate-rich protein [33]. Our demonstration of STEVORs in the merozoite, particularly the rhoptry and an association with invasion-related events provides a foundation for speculating *stevor *multigene family members as potential vaccine target antigens against the invasive forms of the *P. falciparum *life cycle.

## Conclusion

The presence of STEVOR variants in the merozoite and its secretory organelle, the rhoptry were demonstrated. They appear to be associated with erythrocyte invasion events, drawing the attention to an aspect of STEVOR function that may be critical to parasite survival. It is conceivable that these proteins encoded by the *stevor *multigene family are involved in erythrocyte adhesion during invasion or in assisting free merozoites to evade the host immune system and to escape from several of its effector mechanisms.

## Authors' contributions

AK and M–QK conceived and designed the experiments and wrote the manuscript. AK produced the anti-STEVOR antibodies and analyzed the data. IB generated different parasite binding phenotypes, performed the immunoblots and processed the electron microscopic images. AK and IB performed the immunofluorescence assays. NS expressed the recombinant STEVOR proteins. MP purified parasites at the merozoite and gametocyte stages. CS performed the immunoelectron microscopy. All authors discussed the results and approved the final manuscript.
